# One Health Insights From *Pteropus medius*: Nipah Virus Spillover, Microbiota, and Antimicrobial Resistance

**DOI:** 10.1002/mbo3.70362

**Published:** 2026-07-01

**Authors:** Punam Chowdhury, Shah Md Tanvir Khan, Sajal Roy, Md. Shohel Al Faruk

**Affiliations:** ^1^ Department of Physiology, Biochemistry and Pharmacology Chattogram Veterinary and Animal Sciences University (CVASU) Chattogram Bangladesh; ^2^ Department of Biochemistry & Molecular Biology Gopalganj Science and Technology University Dhaka Bangladesh

**Keywords:** antimicrobial resistance, Bangladesh, bat microbiota, Nipah virus, *Pteropus medius*

## Abstract

*Pteropus medius* is a major reservoir of Nipah virus (NiV), a zoonotic pathogen responsible for recurrent fatal encephalitis outbreaks in Bangladesh. Human infections are primarily associated with consuming raw date palm sap contaminated with bat excreta. Beyond viral spillover, growing evidence suggests that bat‐associated microbiota and guano are potential reservoirs of antimicrobial resistance (AMR), contributing to the environmental dissemination of resistant bacteria and resistance genes. This narrative literature review examined the relationship among NiV spillover, bat microbiota, and AMR within a One Health framework. A structured literature search was conducted using PubMed, Scopus, Web of Science, ScienceDirect, and Google Scholar to identify relevant peer‐reviewed studies published between 2006 and 2025. The reviewed evidence indicates that *Pteropus medius* populations harbor clinically important resistant bacteria, including ESBL‐(Extended‐Spectrum Beta‐Lactamase) producing *Escherichia coli*, *Salmonella* spp., and methicillin‐resistant *Staphylococcus* spp. In addition, recent studies indicate that bat roosts near agricultural lands, wastewater discharge sites, and peri‐urban settlements may facilitate bidirectional exchange of ARGs among wildlife, livestock, and humans. Habitat fragmentation, wastewater contamination, urbanization, agricultural intensification, and increased human–wildlife interactions were identified as major drivers facilitating both NiV spillover and AMR dissemination. Overall, the literature demonstrates a significant ecological association among anthropogenic environmental disturbance, zoonotic spillover risk, and the emergence of antimicrobial resistance in bat‐associated systems. These findings highlight the importance of integrated One Health surveillance and environmental management strategies to mitigate future zoonotic and AMR threats in Bangladesh.

## Graphical abstract

Environmental and anthropogenic drivers of antibiotic resistance dissemination. The cycle reinforces how anthropogenic inputs, such as sewage, animal dung, hospital effluent, and agricultural runoff bring resistant bacteria and antibiotic residues into soil and water.

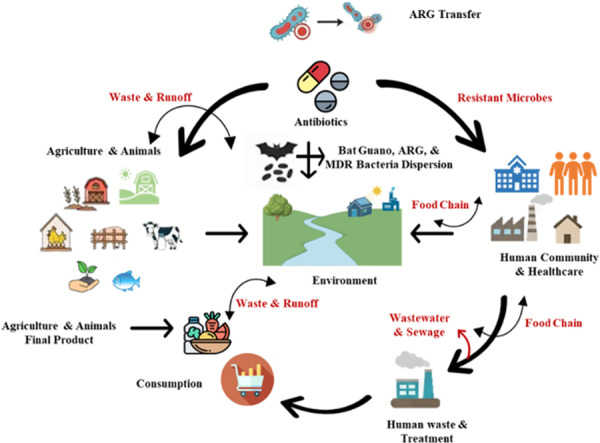

## 1 Introduction

Numerous zoonotic viruses that cause serious illnesses in humans, such as Marburg, Ebola, Nipah, Hendra, SARS, and MERS, have been found to naturally reside in bats. The frugivorous giant bat *Pteropus medius*, also called *P. giganteus* or Indian flying fox, is found throughout Southeast Asia and has been shown to harbor a variety of viruses, including the Nipah virus. Due to its high fatality rate, up to 90% in Bangladesh and India, and its connection to human‐to‐human transmission, the Nipah virus is an emerging zoonotic paramyxovirus that has caught the attention of scientists and public health experts. Despite being highly pathogenic to humans and several other mammals, the virus remains asymptomatic in its natural reservoir host, fruit bats. (Fouret et al. 2020) The ecology and distribution of *Pteropus medius* contribute significantly to its role in zoonotic disease transmission. According to Kryštufek (2009), habitat selection and agricultural land use by bats are strongly influenced by food availability and synanthropic behavior (Kryštufek 2009). Members of the *Pteropodidae* family are highly mobile and frequently inhabit areas close to water bodies, botanical gardens, villages, urban settlements, and human residences. Human–wildlife conflicts in these increasingly anthropogenic landscapes pose risks to ecological stability and public health while also serving as indicators of ecosystem health in tropical regions. Bangladesh is home to two megachiropteran bat species, *Pteropus medius* and *Rousettus leschenaultii*, which belong to the Megachiroptera suborder (Srinivasulu et al. 2010).

*Pteropus medius* is distributed throughout much of South Asia, ranging from eastern Myanmar to Bangladesh, and occupies diverse habitats including mangrove forests of the Sundarbans, deciduous forests, mixed‐evergreen forests, and tropical broadleaf forests. In Bangladesh, the Indian flying fox has been reported from Barisal, Dhaka, Mymensingh, Rajshahi, Rangpur, Sylhet, and Saint Martin's Island (McEvoy et al. 2021). Consumption of raw date palm sap contaminated with bat saliva, urine, or feces is considered one of the major transmission pathways of NiV in Bangladesh. The transmission dynamics of NiV are influenced by bat roosting behavior, climatic conditions, and interactions between wildlife and human populations (Azuero et al. 2023; McKee et al. 2021).

Beyond their role in viral spillover, bats host diverse microbial communities that contribute to the environmental spread of antimicrobial resistance (AMR), while human activities influence both zoonotic spillover processes and the expansion of environmental resistomes (Kumar et al. 2022). Habitat destruction, urbanization, agricultural expansion, and disturbance of bat roosting sites increase interactions among bats, humans, livestock, and contaminated environments, thereby facilitating pathogen transmission. After Nipah virus outbreaks, bat persecution and displacement alter colony movement patterns and contribute to wider viral distribution through relocation. Similarly, environmental contamination from hospital wastewater, livestock manure, agricultural runoff, and antimicrobial residues contributes to the expansion of the environmental resistome by selecting for antibiotic‐resistant bacteria (ARB) and antimicrobial resistance genes (ARGs) (Ahmed et al. 2025; Popoola et al. 2025). Due to its synanthropic behavior, long‐distance foraging capacity, and close association with human‐modified landscapes, *Pteropus* medius functions as an ecological bridge connecting contaminated environments with the spread of resistant microorganisms. These interconnected processes highlight the importance of adopting a One Health perspective to better understand the relationship between zoonotic disease emergence, wildlife ecology, and the environmental spread of AMR.

Bat gastrointestinal tracts harbor diverse microbial communities, and bat guano has emerged as an important non‐invasive source for studying gut microbiota, zoonotic pathogens, and antimicrobial resistance genes (ARGs). Although bat‐associated viral zoonoses are well studied, the role of bats as reservoirs and disseminators of antimicrobial resistance remains less understood. Guano contains several antibiotic‐resistant bacteria, including *Salmonella*, *Enterococcus*, and *Escherichia coli*, highlighting its potential role in the dissemination of AMR. One of the earliest studies from West Java and Krakatau Island, Indonesia, identified dominant Gram‐negative isolates such as *Escherichia coli*, *Klebsiella*, and *Enterobacter*, which showed resistance to ampicillin, trimethoprim, sulphamethoxazole, and cephalothin, with human fecal contamination suggested as a possible source of resistance. Members of the *Enterobacteriaceae* family are particularly significant because some produce extended‐spectrum β‐lactamases (ESBLs), enzymes capable of hydrolyzing third‐ and fourth‐generation β‐lactam antibiotics, including cefotaxime. (Homeier‐Bachmann et al. 2022) Advances in DNA extraction, amplification, and high‐throughput sequencing have further improved guano‐based microbial analyses, consistently revealing bacterial taxa such as *Bacillus*, *Lactococcus*, *Enterobacter*, and *Staphylococcus* across different bat species and regions, reflecting both gut‐associated and environmental microbial influences. For more than two decades, fecal DNA and guano have been widely used to investigate wildlife biology and microbial ecology without disturbing animals. In bats, guano provides an accessible source of genetic material for studying gut microbiota, pathogen surveillance, and ARGs, despite challenges such as degraded DNA and PCR inhibitors. These findings suggest that guano microbiota reflects both bat‐associated gut communities and environmental influences from surrounding roost habitats (Gerbáčová et al. 2020; Banskar et al. 2016b; Newman et al. 2018b). Consequently, guano‐based surveillance has emerged as an important approach for understanding microbial ecology, zoonotic pathogens, and environmental dissemination of antimicrobial resistance in bat‐associated systems (Arandjelovic et al. 2009; Walker et al. 2009).

Although culture‐based studies have provided important insights into bat‐associated bacteria and antimicrobial resistance, they capture only a limited fraction of the total microbial diversity (Abdelfattah et al. 2018). This limitation reduces their ability to represent the full environmental resistome. To address this, high‐throughput sequencing technologies have increasingly been applied to characterize guano‐associated microbial communities more comprehensively (Knight et al. 2018). Sequencing‐based studies of bat guano from geographically distinct regions, including the USA, India, and China, identified dominant taxa such as *Weissella*, *Lactococcus*, *Enterococcus*, *Bacillus*, and *Arthrobacter* (Banskar et al. 2016b; Newman et al. 2018b). These approaches can detect a broader range of antimicrobial resistance genes, including those associated with non‐culturable and low‐abundance microorganisms. However, sequencing data primarily indicate genetic potential rather than confirmed functional resistance, as the presence of resistance genes does not necessarily correspond to expressed or clinically relevant phenotypes. As a result, culture‐based and HTS approaches offer complementary but fundamentally different perspectives, and integrating both is essential for accurately interpreting environmental AMR dynamics.

In addition to zoonotic pathogen transmission, antimicrobial resistance (AMR) has emerged as another major global public health concern associated with environmental and wildlife interfaces (Agustin et al. 2025; Nazwar et al. 2023; Sinto et al. 2024). According to the World Health Organization (WHO), AMR is one of the biggest health issues of the twenty‐first century. (Marshall and Levy 2011) Multidrug‐resistant bacteria are quite prevalent these days and pose a serious threat to both human and animal health. Since resistant bacteria and ARGs spread through shared environments, AMR has become a global rather than a localized issue. South Asian countries, particularly Bangladesh, are considered important hotspots of AMR due to high population density, widespread antimicrobial usage, and extensive human‐animal‐environment interactions. (Kang and Song 2013) Due to their ecological adaptability, migratory behavior, and extensive interactions with diverse environments, bats are considered ecological dissemination hosts that contribute to the environmental dissemination of resistant bacteria and antimicrobial resistance genes (ARGs). Their movement across natural and anthropogenically altered habitats facilitates microbial exchange and promotes the spread of resistance determinants through contaminated environmental sources exposed to bat guano. These characteristics make bats important ecological indicators for understanding the environmental dynamics of AMR transmission. (Henry et al. 2018) Antimicrobial‐resistant fecal bacteria have been identified in diverse wildlife species worldwide, including fish, birds, reptiles, and mammals. Several studies have also detected ARGs and potentially zoonotic bacterial genera in bats. In free‐ranging bats, the prevalence of *Salmonella spp*., *Campylobacter jejuni*, and *Clostridium perfringens* has been reported to be 20%, 19.3%–44%, and 98%, respectively (Hatta et al. 2016; Vengust et al. 2018).

From a One Health perspective, wildlife occupies a critical position at the interface of environmental and public health. Although wildlife species are not typically exposed directly to antibiotic treatment, they may acquire resistant bacteria through contaminated food, water, environmental exposure, or interactions with humans and domestic animals in anthropogenically altered habitats (Łopucki et al. 2024; Meramo et al. 2022) (Tornberg‐Belanger et al. 2022). Wildlife can function as both reservoirs and vectors of resistant bacteria, facilitating the dissemination of resistance across ecosystems and even continents. Therefore, bats represent important model organisms for understanding the ecological dynamics of AMR and for developing effective One Health surveillance strategies to mitigate its environmental spread (Stapelfeldt et al. 2023).

This review aims to provide an overview of the current knowledge regarding antimicrobial resistance patterns associated with *Pteropus medius*, with particular emphasis on fecal microbiota dynamics and their public health implications. Specifically, this review seeks to: (i) evaluate fecal sampling as a practical tool for monitoring pathogen spillover and AMR in wildlife, (ii) describe the genetic determinants of resistance, and (iii) explore ecological and anthropogenic factors influencing transmission dynamics within a One Health framework.

## 2 Methods

### 2.1 Review Design

This review was conducted as a narrative literature review using a structured and comprehensive search strategy to synthesize current evidence on *Pteropus medius*, Nipah virus spillover ecology, bat‐associated microbiota, and antimicrobial resistance within a One Health framework. The review integrates findings from virology, microbiology, ecology, environmental health, and zoonotic disease research to examine the interconnected roles of bats, environmental change, and the dissemination of antimicrobial resistance.

### 2.2 Literature Search Strategy

The electronic databases PubMed, Scopus, Web of Science, ScienceDirect, and Google Scholar were used in a methodical literature search. The search was performed between September 2025 and January 2026 to identify relevant peer‐reviewed publications. Studies published from 2006 to 2025 were considered to ensure the inclusion of both foundational and recent evidence on Nipah virus epidemiology, bat microbiota, and antimicrobial resistance.

Search queries were developed using combinations of Medical Subject Headings (MeSH) and free‐text terms connected through Boolean operators (“AND” and “OR”). The main search terms included: *Pteropus medius* OR *Pteropus giganteus* OR Indian flying fox, Nipah virus OR *Henipavirus*, spillover” OR zoonotic transmission, bat microbiota OR bat gut microbiome OR bat guano microbiome, antimicrobial resistance OR AMR OR antibiotic resistance genes, ESBL‐producing bacteria, wildlife‐associated bacteria, environmental resistome, One Health, bat‐associated zoonoses. The indexing techniques and search specifications of each database were taken into consideration when modifying search strings. Backwards citation monitoring of chosen publications and review papers was used to manually find more relevant works.

### 2.3 Study Selection and Eligibility Criteria

Reviewed articles were screened in two stages. First, titles and abstracts were checked for relevance to the review objectives. Then, full texts of the selected studies were evaluated using predefined inclusion and exclusion criteria. Studies from South and Southeast Asia, especially Bangladesh and India, were prioritized due to the repeated Nipah virus outbreaks and the distribution of *Pteropus medius* in these regions. The inclusion and exclusion criteria, summarized in Table 1, guided the screening and selection of studies. In brief, included studies were research or review articles published after 2006, in English, and covering all Nipah Outbreak regions, whereas nonoriginal publications (books, ad chapters, and conference proceedings), articles published before 2006, or in other languages were excluded.

**Table 1:** The inclusion and exclusion criteria for study selection.

Criterion	Inclusion	Exclusion
Record type	Research articles, review articles, epidemiological studies, molecular studies, surveillance ad case reports.	Books, book chapters, conference proceedings.
Record language	English Articles	Non‐English Articles
Record timeline	Published after 2006	Published before 2006
Record country	South and Southeast Asia	/
Study focus	Studies on *Pteropus* species (especially *Pteropus medius*) related to Nipah virus ecology, spillover, microbiota, antimicrobial resistance, and One Health	Studies focusing only on non‐bat wildlife without relevance to *Pteropus* spp.
Publication quality	Peer‐reviewed journal articles	Duplicate publications or overlapping datasets

### 2.4 Data Extraction and Synthesis

Relevant data were taken from the chosen research and arranged according to subject groups, including:

1. Nipah virus epidemiology and spillover mechanisms;
2. Bat ecology and environmental drivers of transmission;
3. Bat‐associated bacterial diversity and microbiota composition;
4. Antimicrobial resistance profiles and resistance genes;
5. Environmental and anthropogenic factors influencing AMR dissemination;
6. Public health and One Health implications.

The extracted evidence was synthesized narratively to provide an integrated overview of the ecological and microbiological interactions linking zoonotic spillover and antimicrobial resistance in bat‐associated systems. Particular emphasis was placed on the role of habitat disturbance, wastewater contamination, agricultural intensification, and human–wildlife interactions in facilitating both viral emergence and environmental dissemination of antimicrobial resistance.

### 2.5 Quality Appraisal and Limitations

Each included study was critically evaluated with consideration of methodological design, sample representativeness, ecological context, and potential sources of bias. Because the present study was conducted as a narrative review rather than a formal systematic review or meta‐analysis, no standardized risk‐of‐bias assessment tool was applied. Instead, methodological heterogeneity among studies, including differences in sampling strategy, laboratory methodology, antimicrobial susceptibility testing approaches, sequencing techniques, geographic setting, and study duration, was assessed qualitatively to support critical interpretation of the findings. Therefore, resistance prevalence and microbial composition reported across studies should be interpreted cautiously within the context of methodological limitations and environmental variability.

## 3 Ecological and Epidemiological Dimensions of Nipah Virus Spillover

### 3.1 *P. medius* and Nipah Virus Spillover Cases

Nipah virus infection (NiV) is caused by a negative‐sense single‐stranded RNA virus with a length of 18,000 nucleotides belonging to the family *Paramyxoviridae*, genus *Henipavirus*. (Garbuglia et al. 2023; Halpin et al. 2011; Vijayreddy Vandali et al. 2018) Although it does not cause overt disease in bat reservoirs, human case‐fatality rates can reach 40% to 70%. Serological and molecular evidence have detected NiV or antibodies in multiple regions of Asia, including urine, saliva, serum, and tissues of Pteropus bats, indicating widespread circulation. The main reservoir, *Pteropus medius*, is distributed across South and Southeast Asia, where dense human and livestock populations increase spillover risk. Transmission occurs through direct or indirect contact with infected bats or intermediate hosts, particularly via contamination of date palm sap with bat saliva, urine, or feces, a major route in Bangladesh (McKee et al. 2022; Wickenhagen et al. 2024). Human behavior and interactions with bats are the key elements that affect the spread of NiV from bats to people. Climate and habitat loss also have an impact on the virus's shedding and disease transmission to people. The causes of this spillover have not been deeply studied yet. The first recognized human outbreak occurred in Malaysia in 1998 via pigs as intermediate hosts, followed by repeated outbreaks in Bangladesh since 2001 and sporadic cases in India. (Hahn et al. 2014) (Kumar et al. 2022) Between 2012 and 2018, 47 main cases of the Nipah virus, which is a result of bat spillover, were found. According to studies, the primary viral reservoir in Bangladesh and India is the fruit bat *Pteropus medius*. In Bangladesh, spillover incidents have mostly been documented during the winter. This seasonal pattern is thought to be linked to higher consumption of raw palm juice in colder months, as well as increased bat foraging activity and physiological stress associated with thermoregulation during winter. Additionally, habitat loss, urbanization, and climate‐related stress further intensify spillover risk by increasing bat foraging in human‐dominated areas and altering host distribution. Human‐wildlife contact has risen due to the destruction of wild habitats, increasing the likelihood of spillovers. Certain bat species seek out human‐dominated regions for roosting and feeding while wild habitats and forests are being destroyed. They roost close to human homes and take advantage of other resources that people give. (McKee et al. 2022) The major documented spillover events and their epidemiological characteristics are summarized in Table 2.

**Table 2:** Major nipah virus spillover events associated with *pteropus* spp.

Country	Date	Symptoms	Morbidity rate (CFR)	References
West Malaysia (Perak, Negeri Sembilan, Selangor)	September 1998–February 1999	Acute febrile encephalitis, headache, altered consciousness, respiratory distress	~40% (105/265 cases)	Hauser et al. (2021)
Sikamat, Negeri Sembilan (Malaysia)	December 1998–January 1999	Severe encephalitis, fever, and neurological impairment	~40%	
Bukit Pelandok, Negeri Sembilan (Malaysia)	December 1998	Acute encephalitis with respiratory involvement	~40%	
Meherpur, Bangladesh	April 2001	Fever, encephalitis, unconsciousness	~70%	Gurley et al. (2007)
Multiple districts, Bangladesh	2001–present (Seasonal: December–April)	Encephalitis, respiratory symptoms, and occasional person‐to‐person transmission	40%–75% (varies yearly)	Luby et al. (2009); Daszak et al. (2013)
West Bengal, India	2001 and 2007	Acute encephalitis, nosocomial transmission documented	~68%–75%	Gurley et al. (2007)
Kerala, India	May 2018	Severe encephalitis, respiratory failure	~89% (17/19 cases)	Yadav et al. (2022)

### 3.2 Epidemiology of Nipah Virus in Bangladesh

The transmission patterns, clinical symptoms, illness severity, and mortality rates of NiV patients found in Bangladesh are different from those found in other nations. For instance, approximately 28% of NiV‐positive patients in Bangladesh are secondary cases. Clinical manifestations of Nipah virus infection vary across outbreaks in Malaysia, Singapore, Bangladesh, and India. In Singapore, atypical pneumonia was reported in approximately 27% of cases, whereas only around 14% of patients in Malaysia presented with a non‐productive cough. By contrast, outbreaks in Bangladesh (69%) and Siliguri, India (51%) showed a higher frequency of respiratory involvement, alongside neurological symptoms. In terms of severity, Bangladesh has recorded one of the highest case fatality rates at about 71%, compared with Malaysia (53%), the Philippines (29%), and Singapore (21%) (Hegde et al. 2024).

**Figure 1:**
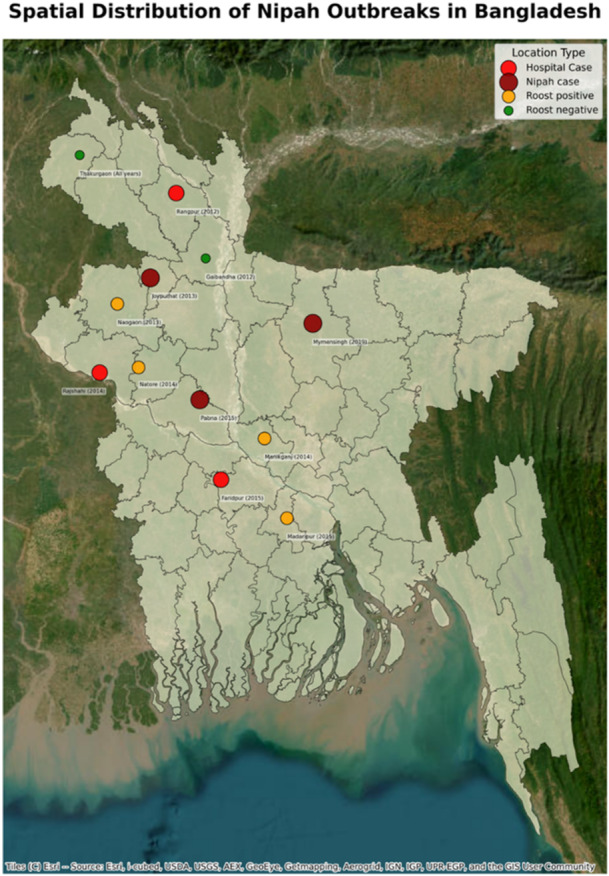
The map shows the Spatial distribution of Nipah virus (NiV) cases (*n* = 21) and bat roost (*n* = 30) surveillance sites in Bangladesh during 2012–2019. Red circles indicate hospital case locations, dark red circles indicate confirmed Nipah cases, orange circles represent NiV RNA‐positive bat roosts, and green circles represent NiV RNA‐negative bat roosts.

According to a study by Clifton D. McKee et al. on Nipah virus detection at roosting sites after spillover events within the timeframe 2012 to 2019, field teams in Bangladesh looked for *P. medius* bat roosts within a 20‐kilometer radius of the human case‐patient's home by scouting and asking locals about known roost locations. A total of 30 bat roosts associated with 21 suspected human cases were identified and sampled. The spatial distribution of the investigated human cases and bat roost surveillance sites across Bangladesh is shown in (Figure 1). The figure illustrates the locations of confirmed and suspected Nipah cases, as well as NiV RNA‐positive and NiV RNA‐negative bat roosts identified during the surveillance period. Teams recorded patient residence locations, symptom onset dates, and estimated exposure times. In some cases, exposure was linked to date palm sap consumption, while in others, it was estimated as 7 days before symptom onset based on the incubation period. Interestingly, seven roosts found close to the patients' residences produced no Nipah virus RNA, and five of the suspected patients tested negative for the virus using PCR or ELISA. The study found no significant association between roost positivity and factors such as distance from patient homes or patient test results. However, roosts with virus‐positive urine samples were more often linked to confirmed human cases and were sometimes located farther from residences and sampled earlier after exposure (McKee et al. 2022).

### 3.3 Ecological Factors Affecting *Pteropus Medius*'s Nipah Virus Maintenance

NiV detection is not limited to the Nipah outbreak regions, indicating that if an appropriate strain and bat‐human interaction were present, viral dissemination can occur anywhere in Bangladesh. There may be localized strains that are continuously circulating in bats if different strains are found in different places, and there is a high degree of homology in one place over time. The main cause of human epidemics in Bangladesh is probably human activities like date palm sap harvesting, along with viral circulation in local bat populations. Moreover, the interaction between intensively managed livestock and the wildlife reservoir *Pteropus* spp. fruit bats led to the emergence of the Nipah virus. The distribution of these infections in their wildlife reservoirs is influenced by several anthropogenic environmental changes, socioeconomic variables, and demographic shifts, all of which contribute to the formation of this and other henipaviruses (Daszak et al. 2013). The fruit bat's native habitat has been significantly reduced due to historical patterns of forest loss. Nowadays, these bats primarily inhabit small resident roosts near people and feed opportunistically on cultivated food sources. These slow but significant alterations have created a system that makes it easier for a virus carried by bats to spread. After consuming fermented or raw date palm juice tainted with Nipah virus‐containing bat excrement, the result is an almost yearly spillover of the virus during the winter. The ecology of the Nipah virus in Bangladesh and the related Hendra virus in *Pteropus* spp. in Australia have significant parallels and contrasts. In Bangladesh and Australia, bat spillover events mostly take place during the colder, dry winter months. Research from Australia indicates that during this time, bats are under nutritional stress, live in small roosts near people, and shed more viruses.

The ecological determinants of Nipah virus differ between Bangladesh and Malaysia, although both settings show that human‐altered landscapes and changes in resource use have contributed to spillover events in humans. In Malaysia, the Nipah virus circulated in *Pteropus* spp. and was first transmitted to domestic pigs, where it caused respiratory disease and facilitated onward transmission to humans through respiratory droplets. Analyses of long‐term agricultural data suggest that increased commercial pig farming and mixed farming systems created conditions that allowed repeated transmission of the virus from bats to pigs. In addition, the initial introduction of the virus enabled its persistence within pig populations, increasing subsequent transmission between pigs and to humans. Bangladeshi Nipah virus outbreaks follow a completely distinct ecological trend. In Bangladesh, outbreaks are seasonal, with instances mostly concentrated in the country's northwest and central regions between December and April. Unlike the Malaysian context, where pigs acted as intermediate hosts (McKee et al. 2022), transmission in Bangladesh is mainly foodborne. Bat visits to date palm trees are most frequent during winter (Islam et al. 2023) (October–April), which coincides with the peak season for sap harvesting and reduced availability of alternative fruit resources (McKee et al. 2021).

### 3.4 Mechanisms of Virus Spillover From Bats to Humans and Livestock

Prior research has demonstrated that human behavior and activity are the primary causes of viral shedding events. The main causes appear to be anthropogenic alterations that facilitate interactions between humans and wildlife, which are further exacerbated by elements that affect viral shedding in host wild animals. NiV spreads from its natural reservoir in *Pteropus* bats to humans and cattle through a number of pathways that frequently overlap and are based on behavioral, ecological, and epidemiological dynamics. Direct contamination of human food or raw date palm sap is one of the main pathways in Bangladesh. Bangladesh is home to *P. medius*, and throughout the year, bats excrete the Nipah virus in their urine. At 22°C, the virus can still remain infectious in bat urine with a neutral pH for a maximum of 4 days.

Another crucial route is indirect spillover through intermediary animal hosts. In six locations where spillover human NiV infection cases occurred between 2013 and 2015, serological research has shown signs of NiV exposure in peridomestic animals such as dogs, cats, and. This suggests that, due to their unrestricted movement, these animals potentially scavenge bat carcasses or placental material beneath roosting sites without human awareness. Previous studies have shown cases of henipavirus infection in cats and dogs in several countries. Identifying the factors that increase infection risk, along with the possible role of domestic animals as intermediate or amplifying hosts, can provide a more comprehensive understanding of NiV ecology in Bangladesh. Local NiV transmission in bat populations occurs year‐round, according to modeling and field data, and is caused by density‐dependent transmission, gradual immunity loss, and potential viral recrudescence (i.e., reactivation of latent infection). Periodic spikes in viral shedding may result from these multi‐annual cycles of declining immunity and bat population turnover, raising the possibility of transmission. Temporal shedding of NiV has been discovered through monitoring bat roosts near human cases. For instance, even weeks after the suspected human exposure, viral RNA was found in pooled bat urine taken from roosts close to human infections, but the positivity quickly decreased over time. This implies that shedding risk fluctuates over time and geography and is correlated with spillover events (Islam et al. 2023; Epstein et al. 2020; McKee et al. 2022).

### 3.5 Anthropogenic Drivers and Environmental Resistance

Bats serve as crucial reservoirs for several new zoonotic viruses, such as the Nipah virus, Ebola virus, Marburg virus, SARS‐related coronaviruses, and Hendra virus. Their biological variety, mobility, and adaptability to anthropogenic contexts make them significant in disease transmission. The central question in wildlife‐associated AMR research is whether bats act as primary evolutionary sources of ARGs or as ecological sinks acquiring resistant bacteria from anthropogenically impacted environments. Current evidence more strongly supports the “sink” hypothesis, with bats mainly serving as secondary hosts and dissemination agents rather than sources of clinically relevant resistance determinants. ARGs such as blaCTX‐M, blaTEM, and blaSHV detected in bats are commonly associated with hospital wastewater, livestock systems, sewage, and agricultural runoff, indicating a strong anthropogenic influence (Devnath et al. 2023; Luo et al. 2020; Kuralayanapalya et al. 2019). Higher AMR diversity and multidrug‐resistant bacterial carriage are consistently reported in bats from urban and agricultural landscapes compared to less disturbed habitats, and similarity in resistance profiles between bats and nearby livestock further supports shared environmental exposure pathways (Ahmed et al. 2025; Berendonk et al. 2015; Henry et al. 2018; Martinez 2009; Tornberg‐Belanger et al. 2022). Environmental disturbance plays a key role in shaping both viral and antimicrobial resistance dynamics. Similarity of resistance profiles between bats and nearby livestock or human‐associated bacteria further supports shared environmental exposure rather than independent evolution. (Devnath et al. 2023; Luo et al. 2020). A recent study indicates that spillover is affected not just by viruses but also by ecological disturbances, microbial interactions, and changes in the environment (Letko et al. 2020). In South and Southeast Asia, the emergence of the virus is closely associated with fruit bats of the genus *Pteropus medius*. In Bangladesh, land‐use change, deforestation, urban expansion, and increasing human encroachment into bat habitats have altered bat foraging and roosting behavior, increasing interactions among bats, livestock, and humans. These changes elevate the risk of zoonotic transmission and also contribute to environmental dissemination of antimicrobial resistance genes (McKee et al. 2021).

Human responses to outbreaks can further intensify ecological disruption. Bat culling and roost destruction following Nipah outbreaks may force relocation of colonies, potentially altering viral circulation patterns and increasing contact with new environments (Kumar et al. 2022). At the same time, anthropogenic activities strongly shape the environmental resistome, which refers to the total collection of ARGs in microbial communities.(Lee et al. 2020) Exposure to wastewater, sewage, animal waste, agricultural runoff, aquaculture effluents, and pharmaceutical contamination promotes the acquisition of resistant bacteria and ARGs, which may integrate into bat gut microbiota and drive long‐term resistome shifts (Berendonk et al. 2015; Zhai et al. 2025). Bats using polluted water may get resistant microorganisms, which then integrate into their gastrointestinal microbiome, leading to long‐term resistome rearrangement (Berendonk et al. 2015; Martinez 2009). Although direct mechanistic links between Nipah virus dynamics and ARG acquisition remain unclear, environmental disturbance is strongly associated with changes in bat microbiota and increased interspecies microbial exchange (Luis et al. 2026; Plowright et al. 2026). Due to their high mobility and wide feeding ranges, bats act as both recipients and spreaders of antimicrobial resistance and viral infections between different ecological environments.

Within this context, the Indian flying fox represents an important ecological interface linking anthropogenic environments with wildlife‐associated microbial communities. Due to its colonial roosting behavior, long‐distance foraging, and synanthropic adaptation, *P. medius* is frequently exposed to anthropogenically contaminated environments across urban, peri‐urban, and agricultural landscapes (Ahmed et al. 2025; Roy et al. 2024). Studies have linked land‐use patterns around bat roosts with increased detection of antimicrobial‐resistant bacteria in fecal samples, including resistance to critically important antimicrobials such as aztreonam and gentamicin. The frequent detection of β‐lactam resistance genes in extended‐spectrum β‐lactamase (ESBL)‐producing *Escherichia coli* further supports environmental acquisition of ARGs mediated by mobile genetic elements such as plasmids, transposons, and integrons. Bat guano also contributes to environmental dissemination by redistributing resistant bacteria and ARGs into soil, water, and agricultural systems, reinforcing ecological connectivity between wildlife and human‐associated environments. Similarities between resistance profiles observed in bats and nearby domestic animals support the hypothesis of shared environmental exposure pathways rather than independent resistance evolution. Collectively, these findings emphasize the importance of including synanthropic bat species such as *P. medius* in AMR surveillance frameworks, as their ecological interactions can contribute to the environmental circulation and cross‐ecosystem dissemination of resistant bacteria and antimicrobial resistance genes (Ahmed et al. 2025).

### 3.6 Environmental Disturbance, Bat Microbiota, and Spillover Dynamics

Although numerous studies associate habitat disturbance, urbanization, and pollution with increased zoonotic risk, causal relationships remain difficult to establish due to the predominantly observational or cross‐sectional nature of current research (Festa et al. 2023; Phelps and Kingston 2018; Sherwin and Sciences 2012). In this context, the bat microbiome acts as a key but still underexplored mediator linking environmental change with host health and pathogen dynamics.

Bat microbiota consists of bacteria, fungi, archaea, and viruses that collectively influence immunity, metabolism, and disease resistance. Alterations in microbial composition (dysbiosis) may affect pathogen colonization and viral persistence (Federici et al. 2022; Laboratories 2012). At the molecular level, plasmids, transposons, and integrons facilitate horizontal gene transfer, which is a key way that ARG spreads through environmental and host‐associated bacteria. (Partridge et al. 2018). This change in bats' microbiomes may have even more effects on gut equilibrium and inflammation signals (Turner 2009). In parallel, changes in the immune system involving interferon reactions, interferon‐stimulated genes (IFNs), such as IFNsα, β,ω,κ, and λ, and pro‐inflammatory cytokines (IL8, TNF‐α, and IL1β,) have been linked to controlling how long the virus stays in bat hosts (Banerjee et al. 2020; Irving et al. 2021). It is crucial to note that antibiotic pressure has the potential to favor the co‐evolution of antibiotic resistance genes (ARGs) and virulence traits, which can render microorganisms more fit and more hazardous (Pal et al. 2015).

**Figure 2:**
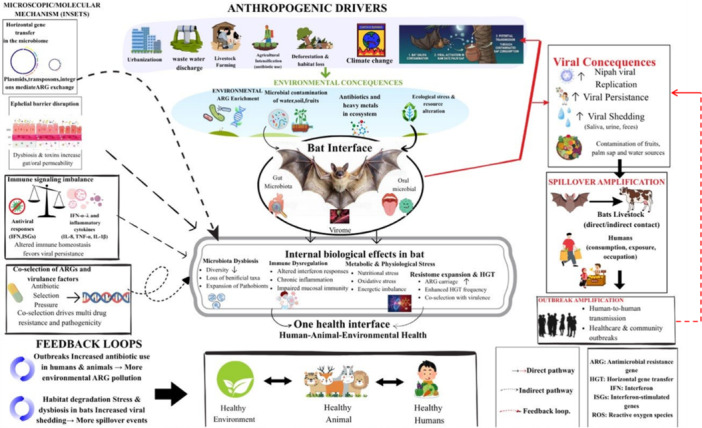
Conceptual framework illustrating anthropogenic drivers, microbiota‐mediated mechanisms, antimicrobial resistance dissemination, and viral spillover amplification at the bat–human–environment interface within a One Health framework. Human‐driven environmental changes and climate change promote environmental contamination with antimicrobial resistance genes, antibiotics, heavy metals, and pathogenic microorganisms. These pressures influence the bat microbiome and virome, resulting in microbiota dysbiosis, immune dysregulation, metabolic stress, and increased horizontal gene transfer (HGT), which collectively facilitate ARG acquisition and persistence. At the microscopic level, microbiome disruption, epithelial barrier impairment, immune signaling imbalance, and co‐selection mechanisms contribute to resistance development. These biological alterations may enhance viral replication, viral shedding, and environmental contamination, increasing opportunities for pathogen transmission between bats, livestock, and humans. Subsequent spillover and outbreak amplification can contribute to broader public health impacts. Feedback loops between environmental contamination, antimicrobial use, habitat degradation, and disease transmission further reinforce AMR dissemination and zoonotic risk.

The interrelated processes are best comprehended under a One Health paradigm (Figure 2), which illustrates the complex interactions among anthropogenic environmental pressures, microbial ecology, antimicrobial resistance dissemination, bat health, and zoonotic transmission pathways linking environmental, animal, and human health. Integrated surveillance strategies that include ecological monitoring, microbiome analysis, resistome characterization, and viral surveillance may boost the prediction capability for Nipah virus spillover and other emerging zoonoses (Plowright et al. 2017).

Overall, the available data indicate that human‐caused environmental stressors such as habitat fragmentation, nutritional stress, and exposure to contaminated environments can influence bat immunity, microbiota composition, and pathogen dynamics. However, these relationships are complex, context‐dependent, and primarily associative rather than causally proven. Consequently, bats are best understood as part of dynamic ecological systems where environmental change shapes both zoonotic transmission risk and antimicrobial resistance dissemination.

## 4 Antimicrobial Resistance in Bat

### 4.1 Antibiotic Resistance Mechanisms

Bats are increasingly recognized as important environmental reservoirs of ARB and ARGs, largely because of their mobility, long lifespan, social roosting behavior, and frequent interactions with anthropogenic environments. Bat‐associated microbiota may acquire resistance determinants through environmental exposure to antibiotics, contaminated water, livestock waste, agricultural runoff, and human‐associated microbial communities (Soto‐López et al. 2025; Kim and Cha 2021b).

Both spontaneous mutations in genes and horizontal gene transfer (HGT) contribute to the development of antibiotic resistance in bacteria linked to bats (Khameneh et al. 2016; Vázquez‐Laslop and Mankin 2018). Among HGT mechanisms, plasmid‐mediated conjugation plays a particularly important role in disseminating ARGs across bacterial populations. Mobile genetic elements (MGEs), including plasmids, transposons, and integrons, facilitate the transfer of multidrug resistance determinants in bat microbiomes. Class 1 integrons carrying ARGs have been identified in flying foxes (*Pteropus poliocephalus*), highlighting bats as reservoirs and dispersal hosts of clinically relevant resistance determinants (McDougall et al. 2019; Soto‐López et al. 2025; Kim and Cha 2021b).

Several major resistance mechanisms have been reported in bacteria isolated from bats. Reduced membrane permeability and alterations in porin are common in Gram‐negative bacteria, limiting antibiotic uptake. Efflux pumps belonging to the ABC, MFS, and RND families actively expel antimicrobial compounds, thereby contributing to multidrug resistance. Metagenomic studies of bat guano have identified genes such as *acrB*, *emrR*, and *qacG*, which are associated with active efflux and resistance to clinically important antibiotics (Soto‐López et al. 2026).

Enzymatic inactivation is another widespread mechanism in bat‐associated bacteria. β‐lactamases are among the most abundant ARGs detected in bat resistomes and confer resistance by hydrolyzing the β‐lactam ring of penicillins and cephalosporins. Aminoglycoside‐modifying enzymes that mediate acetylation, phosphorylation, or adenylation have also been reported in bat‐associated bacterial communities. Recent metagenomic analyses demonstrated that β‐lactamase genes are highly prevalent in bat colonies exposed to anthropogenic environments, suggesting strong environmental selection pressure (Munita and Arias 2016).

Target modification further contributes to resistance in bat‐associated microorganisms. Alterations in penicillin‐binding proteins (PBPs), ribosomal subunits, DNA gyrase, and cell membrane structures reduce antibiotic binding efficiency. Resistance mechanisms involving lipid A modification and altered peptidoglycan biosynthesis have also been detected in environmental resistomes associated with wildlife microbiota (Lin et al. 2015; Peterson and Kaur 2018).

**Figure 3:**
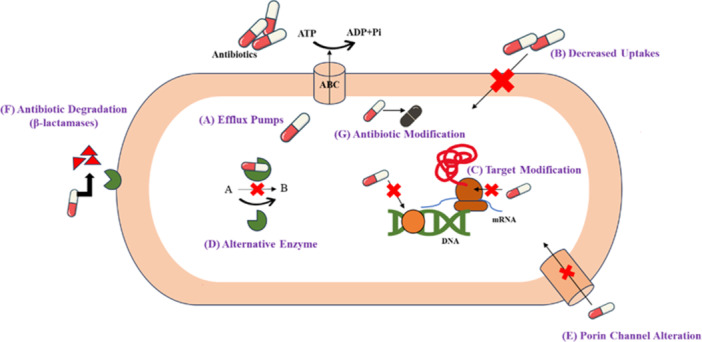
Diagrammatic illustration of various bacterial antibiotic resistance processes. (A) Antibiotic efflux pumps use proton gradients in MFS, MATE, SMR, and RND family pumps or energy from ATP hydrolysis in ABC pumps like DrrAB, OtrC, TlrC, and MlbYZ to remove the antibiotic from the cell. (B) Modifications to the cell membrane that restrict antibiotic entrance result in decreased absorption. (C) Target modification encompasses a variety of target modifications, such as methylation of 23S or 16S rRNA, modifications to peptidoglycan precursors (like glycopeptides), or the synthesis of alternative low‐affinity targets (PBPs) that lessen or totally prevent antibiotics (like penicillins) from binding to the target. (D) By catalyzing the identical reaction with a resistant form, alternative enzymes avoid antibiotic‐inhibited pathways. (E) Changes in the outer membrane porin channel prevent different antimicrobials from entering the cell; (F) β‐lactamases hydrolyze the antibiotic, causing antibiotic degradation. (G) N‐acetyl transferases (AAC), O‐phosphotransferases (APH), and O‐adenyltransferases (ANT) add acetyl, phosphate, or adenyl groups to aminoglycosides to modify antibiotics. Bleomycin N‐acetyltransferases (BlmB) and chloramphenicol acetyltransferases (CAT) are two other examples. (G) Target bypass entails the creation of extra antibiotic targets or subunits that the antibiotic cannot attach to.

Biofilm formation is another important adaptive strategy that enhances bacterial persistence within bat microbiomes. Biofilms protect bacterial communities from antimicrobial exposure and facilitate the exchange of ARGs within dense microbial populations. This mechanism may increase the environmental stability and dissemination potential of resistant bacteria shed through bat feces and saliva (Soto‐López et al. 2025; Kim and Cha 2021b). The principal mechanisms by which bacteria acquire and express antibiotic resistance are summarized in (Figure 3).

Recent shotgun metagenomic studies have shown that anthropogenic habitats harbor a more diverse and clinically relevant bat resistome compared to pristine environments. Bat colonies living near urban or agricultural regions exhibit higher abundances of ARGs associated with β‐lactam resistance, multidrug efflux systems, quinolone resistance, and disinfectant tolerance. These findings support the growing concern that bats may contribute to the environmental circulation of ARGs within a One Health framework linking wildlife, humans, livestock, and ecosystems (Huang et al. 2026).

### 4.2 Evidence of Gram‐Negative Bacterial Pathogens Resistant to Antibiotics in Bats

Evidence of AMR in Gram‐negative bacteria isolated from bats, particularly *Escherichia coli*, *Enterobacter*, and *Salmonella* spp. shows marked geographical heterogeneity, host‐dependent variation, and strong influence of sample size and study design, limiting direct comparability in different studies. Early evidence from Indonesia reported resistance in bat‐associated *E. coli* to trimethoprim (7%), cephalothin (20%), and sulfamethoxazole (27%) (Devnath et al. 2023). However, this study is constrained by its older methodology and limited isolate numbers, making it difficult to contextualize within modern AMR frameworks. In contrast, a Japanese study (2014; *n* = 26 *E. coli* isolates) reported very low resistance profiles, with only minor resistance to streptomycin (3.8% for chlorotetracycline and streptomycin). The relatively small sample size in Japan (*n* = 26) suggests that these findings reflect local ecological conditions or limited colonization rather than the general absence of resistance.

More variable and concerning patterns emerge in African and South American datasets. In Nigeria, multiple studies reported significant resistance to cephalosporin, although isolate numbers were not consistently reported, reducing interpretability. Brazilian studies provide larger datasets, including hundreds of *E. coli* isolates, where resistance patterns were mixed: low resistance to critical drugs such as imipenem and gentamicin was observed, but high resistance to ampicillin (57%) and amoxicillin (54%) was consistently reported across bat oral and excreta isolates. A smaller Brazilian dataset (*n* = 17 *E. coli*) similarly showed ⁓59% ampicillin resistance, reinforcing β‐lactam resistance as a recurring trait in bat‐associated enteric bacteria.

Of particular concern are reports of ESBL‐producing *E. coli* in Peruvian vampire bats (*Desmodus rotundus*), where isolates showed multidrug resistance to multiple β‐lactams, including cefotaxime and ticarcillin. Although the number of isolates was limited, the presence of plasmid‐mediated *blaCTX‐M* genes suggests horizontal gene transfer and environmental exposure to anthropogenic AMR reservoirs rather than bat‐specific selection. In Trinidad, a relatively larger dataset (*n* = 49 *E. coli* isolates) showed high resistance to erythromycin (71%) and streptomycin (26%), although interpretation is complicated by erythromycin's limited efficacy against Gram‐negative bacteria (Devnath et al. 2023).

For *Enterobacter* spp., resistance patterns were similarly heterogeneous. A Brazilian study (*n* = 20) showed high resistance mainly to ampicillin and amoxicillin (> 80%), while a Gabon study (Nguema et al. 2020) reported broad multidrug resistance across β‐lactams and cephalosporins, indicating potential regional hotspots of resistance dissemination.

Reports of *Salmonella* spp. remain sparse and inconsistent. Trinidad isolates showed extreme resistance to streptomycin (100%) and erythromycin (75%), but other regions, such as Bangladesh and Australia, reported fully susceptible isolates. Brazilian isolates showed moderate resistance to ampicillin and cephalexin (⁓50%), but small sample sizes limit epidemiological inference.

Overall, across studies, β‐lactam resistance (particularly ampicillin and amoxicillin) emerges as the most consistent pattern, while resistance to carbapenems and gentamicin remains relatively rare but not absent. Importantly, the detection of ESBL genes across multiple bat species and dietary guilds (frugivorous, insectivorous, hematophagous) indicates that bats function as ecological reservoirs or temporary carriers of anthropogenically derived resistance genes, rather than primary sources of their evolution. (Devnath et al. 2023; Luo et al. 2020; Kuralayanapalya et al. 2019) However, the overall interpretation is constrained by uneven sampling effort (ranging from *n* = 17 to hundreds of isolates) and inconsistent antibiotic panels across studies. This makes it difficult to quantify true global prevalence or compare resistance burdens across regions.

### 4.3 Evidence of Gram‐Positive Bacterial Pathogens Resistant to Antibiotics in Bats

The management of infectious diseases continues to face significant challenges due to AMR in Gram‐positive bacteria (Doernberg et al. 2017). Compared to Gram‐negative bacteria, evidence on Gram‐positive antimicrobial‐resistant bacteria in bats remains limited, yet it reveals an emerging pattern of opportunistic colonization by clinically relevant taxa rather than well‐characterized resistance reservoirs. Studies have primarily reported *Staphylococcus*, *Streptococcus*, *Enterococcus*, and occasionally environmental genera such as *Bacillus* and *Arthrobacter*, isolated from fecal, guano, oral, and occasionally reproductive tract samples.

Two Nigerian investigations carried out in 2012 and 2018 isolated 19.1% and 11.2% of *S. aureus Eidolon helvum* bat fecal samples (Akobi et al. 2012; Olatimehin et al. 2018). However, both studies reported no MRSA (Methicillin‐resistant *Staphylococcus aureus*) detection and only low‐level penicillin resistance, suggesting limited clinical resistance expansion within these populations at the time of sampling.

Multiple *Staphylococcus* species—including *S. aureus, S. kloosii*, *S. sciuri*, and *S. xylosus* have been isolated from insectivorous and frugivorous bats across Slovakia, Spain, and the UK. A Slovak study reported *Staphylococcus nepalensis* from bat guano based on a very small number of isolates (*n* = 5). Later reports noted vancomycin resistance in this species, which is important from a clinical perspective (Marshall and Levy 2011). However, because the evidence is based on a few isolates, it is unclear whether this resistance is consistently present in bats or occurs only as an occasional, acquired trait. Resistance profiles among European *Staphylococcus* isolates generally show widespread resistance to older antibiotics such as erythromycin, streptomycin, and tetracycline, with some studies reporting high or universal resistance rates. However, these patterns are strongly influenced by non‐standardized susceptibility panels and inconsistent reporting thresholds, reducing comparability across studies.

Less commonly reported Gram‐positive taxa provide additional but highly variable signals. A bat‐associated *Bacillus anthracis* report indicated partial resistance to ciprofloxacin and tetracycline (25%) and high resistance to ofloxacin (75%). Similarly, *Arthrobacter* spp. showing partial resistance to vancomycin and chloramphenicol (50%) likely reflects environmental Gram‐positive background flora rather than host‐adapted pathogens.

**Figure 4:**
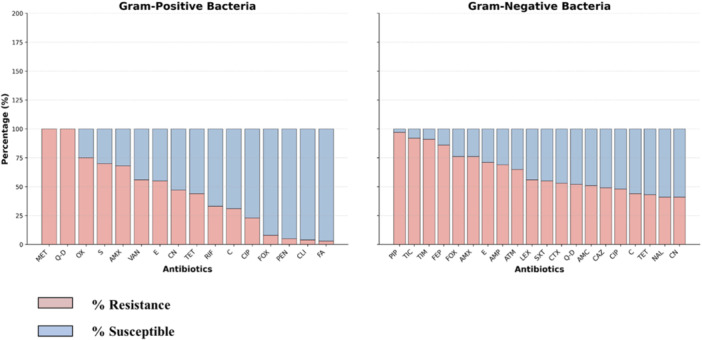
Combined Antibiotic Resistance Profile in Bat (Gram Negative & Gram Positive). Piperacillin = PIP, Ticarcillin= TIC, Ticarcillin‐clavulanic acid = TIM, Cefepime = FEP, Cefoxitin = FOX, Amoxicillin = AMX, Erythromycin = E, AMP = Ampicillin, Aztreonam = ATM, Cephalexin = LEX, Cefotaxime = CTX, Quinupristin‐dalfopristin = Q‐D, Amoxicillin‐Clavulanic acid = AMC, Ceftazidime = CAZ, Ciprofloxacin = CIP, Chloramphenicol = C, Tetracycline = TET, Nalidixic acid = NAL, Gentamicin = CN, Methicillin = MET, Oxacillin = OX, Streptomycin = S, Vancomycin = V, Erythromycin = E, Gentamicin = CN, Tetracycline = TET, Rifampicin = RIF, Chloramphenicol = C, Cefoxitin = FOX, Penicillin = PEN, Clindamycin = CLI, Fusidic acid = FA.

More consistent and epidemiologically relevant evidence comes from *Enterococcus* spp. A Polish study reported *Enterococcus faecalis* from bat guano with high resistance to tetracycline (69.4%), streptomycin (41.7%), and kanamycin (38.9%) (Nowakiewicz et al. 2020), indicating multi‐drug resistance in enteric Gram‐positive commensals with strong environmental antibiotic exposure signatures. Similarly, a Spanish study recovered *Enterococcus* isolates from bat rectal specimens, with resistance to erythromycin and ciprofloxacin in one isolate and universal resistance to quinupristin–dalfopristin, suggesting acquisition of resistance traits common in human‐associated enterococci (Devnath et al. 2023). Figure 4 shows the combined average percentage of significant antibiotic resistance in all Gram‐positive and Gram‐negative bacteria isolated from bats.

Overall, Gram‐positive AMR patterns in bats are characterized by dominance of opportunistic or environmental taxa rather than obligate pathogens, frequent resistance to older, widely used antibiotic classes (tetracyclines, macrolides, aminoglycosides), and limited but concerning detection of clinically critical resistance traits such as vancomycin resistance in isolated cases. However, interpretation is constrained by very small and uneven sample sizes (e.g., *n* = 5 in early *Staphylococcus* reports) and inconsistent antimicrobial testing panels. Unlike Gram‐negative bacteria, where ESBL production and plasmid‐mediated resistance dominate the narrative, Gram‐positive bat‐associated AMR appears to reflect ecological spillover from environmental and anthropogenic antibiotic reservoirs into commensal microbiota, rather than sustained pathogen‐driven resistance amplification within bat hosts.

### 4.4 AMR in *Pteropus medius*

A cross‐sectional study analyzing 369 fecal samples from *P. medius* found that 7.9% of the bats carried AMR *Salmonella* spp. Among the isolates, high resistance was observed against tetracycline (93%), nalidixic acid (86%), and sulfamethoxazole‐trimethoprim (80%) (Rumi et al. 2025). AMR and environmental exposure hazards during various seasons were highlighted in another cross‐sectional investigation. According to the study, none of the *E. Coli* isolates were pandrug resistant (PDR), one isolate was extensively drug resistant (XDR), and 46% of the isolates were multidrug resistant (MDR). Seasonal variations were observed in resistance to many antibiotics, such as cefradine, ampicillin, ceftazidime, azithromycin, and aztreonam. Important human‐use reserve antibiotics, such as imipenem and aztreonam, were also found to have resistance. Winter had a greater rate of aztreonam resistance (17.1%) than summer (4.4%). Furthermore, resistance to Highest Priority Critically Important Antimicrobials (HPCIAs), such as ciprofloxacin and cefotaxime, was noted. The study also revealed that 37% of the *E. coli* isolates had ampicillin resistance and 37% had ESBL‐producing traits. This resistance level was lower than that reported in insectivorous bats from Poland (66%) but higher than findings from Brazil (5%) and Australia (4%) (McDougall et al. 2021). Seasonal differences in resistance patterns further suggest that environmental exposure, feeding behavior, habitat use, and contact with contaminated sources change throughout the year and influence the dissemination of AMR.

The very high resistance to rifampin in *E. coli* isolates is not unexpected because rifampin resistance is mainly caused by mutations in the *rpoB* gene, which are commonly found in environmental bacteria. However, since rifampin is mainly used to treat tuberculosis and is not normally used to treat *E. coli* infections, the clinical importance of this resistance remains unclear. The study also found that azithromycin resistance was more common in winter (22%) than in summer (15%), although azithromycin is not routinely recommended for *E. coli* atibiogram testing according to CLSI guidelines (Kournoutou and Dinos 2022; Mustafa et al. 2021; Rodríguez‐Verdugo et al. 2020). Reported resistance prevalence among *Pteropus medius* populations should be interpreted with caution because cross‐sectional studies provide only a temporary snapshot of resistance prevalence and may not accurately reflect long‐term ecological dynamics. In addition, the origin of resistant bacteria and resistance genes was not identified, preventing a clear determination of whether bats act as reservoirs that disseminate AMR or simply acquire resistant bacteria from contaminated environments. The study also relied mainly on phenotypic resistance testing without detailed molecular characterization, limiting understanding of the specific resistance genes, mobile genetic elements, and mechanisms involved in resistance transfer. Therefore, long‐term longitudinal surveillance combined with genomic and molecular analyses is necessary to better understand the ecological role of bats in AMR dissemination and the associated public health risks.

## 5 Bat Fecal Microbiota

### 5.1 Bacteria and Enteric Pathogens in Bats

Knowledge on the bat bacteriome is still quite limited. Research has shown that bacterial species have a comparatively high degree of diversity and function (Gerbáčová et al. 2020; Nguema et al. 2020). According to a number of studies, the most common bacterial group in the *Chiroptera* order appears to be *Gammaproteobacterial*, with the *Enterobacteriaceae* family predominating, but *Fusobacteria* are less common than in other mammal species. Season, sex, diet type, reproductive stage, and environmental conditions are some of the extrinsic and intrinsic elements that will affect the bacteriome (Gerbáčová et al. 2020). In addition to commensal bacterial strains, strains of human and animal pathogen‐like bacteria have been found in the gastrointestinal flora of individuals and colonies of various bat species. Pathogenic bacteria like *Salmonella, Shigella, Yersinia*, and *Campylobacter* are found in bats (Mühldorfer 2013). Although these bacteria often do not infect bats, they can infect humans and animals and cause illnesses such as meningitis, septicemia, and diarrhea.

Various countries have isolated *Salmonella* spp. from bats; however, their prevalence and serotypes vary, and not all populations are infected. Certain bat serotypes have been linked to animals and humans. Both domesticated and free‐flying birds have been found to harbor *Campylobacter* species, which are widely regarded as significant reservoirs. In contrast to birds that fly freely, little is known about the pathogen that causes bat sickness. Additional *Enterobacteriaceae* family members have been found in bat feces or digestive contents and have been isolated from bat feces and intestinal contents in a number of investigations. Certain of these intestinal bacteria have unusual characteristics related to the foods they consume (Adesiyun et al. 2009). Besides that, many viruses, germs, parasites, and fungi that can cause illness in humans or other animals are found in bats. In addition, many pathogens, some of which are zoonotic, may be stored in bats and their guano. Some zoonoses associated with bats are shown in Table 3.

**Table 3:** Overview of bat‐associated pathogens with zoonotic potential.

Pathogen		Disease caused	References
Bacteria	Campylobacter spp.	Campylobacteriosis	Garcês (2022) and Vashi et al. (2010)
	Mycobacterium spp.	Tuberculosis	
	Salmonella spp.	Salmonellosis	
	Pasteurella spp.	Systemic Infections	
	Brucella spp.	Brucellosis	
	Coxiella burnetii spp.	Q Fever	
	Neoricketssia spp.	Salmon poisoning disease	
	Bartonella spp.	Cat Scratch Disease	
	Borellia spp.	Endocarditis	
	Yersinia spp.	Plague	
	Shigella spp.	Bacillary Dysentery	
	Leptospira spp.	Systemic Infections	
Fungi	Histoplasma capsulatum	Histoplasmosis	Garcês (2022) and Sandoval‐Denis et al. (2013)
	Coccidioides immitis	Coccidioidomycosis	
	Candida albicans	Candidiasis	
	Paracoccidioides brasiliensis	Paracoccidioidomycosis	
	Cryptococcus neoformans	Cryptococcosis	
	Blastomyces dermatitidis	Blastomycosis	
Virus	Rabies virus	Acute fatal encephalitis	Johnson et al. (2010)
	Ebola virus	Ebola hemorrhagic fever	Olival and Hayman (2014)
	Marburg virus	Marburg hemorrhagic fever	
	Irkut virus	Acute fatal encephalitis	Banyard et al. (2014)
	Duvenhage virus	Acute fatal encephalitis	
	Australian Bat Lyssavirus	Acute fatal encephalitis	Weir et al. (2014)
	SARS‐CoV	Severe Acute Respiratory Syndrome	Drexler et al. (2014)
	MERS‐CoV	Middle Eastern Respiratory Syndrome	
	Nipah virus	Severe encephalitis	Clayton et al. (2013)
	Hendra Virus	Fatal respiratory disease	
	Kysanur Virus	Kysanur forest disease	
	Chikungunya Virus	Fever and joint pain	Garcês (2022)

### 5.2 Fecal Microbiota Studies in *Pteropus* spp

Related investigations on *Pteropus giganteus* (now known as *Pteropus medius*) have revealed fecal microbiomes enriched in genes that metabolize carbohydrates and have occasionally detected AMR determinants, despite the paucity of data on *P. medius. Alcaligenes* and *Pseudomonas* were found in the guano of the Indian flying fox, according to a bacteriological analysis. Additionally, the most prevalent fungi in guano were *Fusarium* and *Penicillium*, whereas *Streptomyces* was a prominent actinomycete. Tayiba et al. identified eight fungal taxa from Indian flying fox guano in a different investigation. *Trichophyton, Histoplasma*, and *Cryptococcus* were found in guano samples. The fungal taxa in the *P. giganteus* guano samples also showed seasonal changes. Eight genera were distinguished from guano during the four seasons. *Scopulariopsis* and *Trichophyton* were isolated in the summer and fall, respectively, while only *Exophiala* and *Histoplasma* were isolated in the spring. *Chrysosporium* and *Cryptococcus* were isolated in the summer and winter, while *Alternaria* and *Aspergillus* were present in the spring, fall, and winter. The number of fungal colonies from guano was highest in spring (4.0 × 10^4^ CFU/gm) and lowest in autumn (3.0 × 10^4^ CFU/gm). (Gulraiz et al. 2017)

Additionally, *Acaligens, Azotobacter, Bartonella, Nitrosomonas, Pseudomonas*, and *Salmonella* were the six genera that were isolated from guano. Researchers have discovered the most current lineage D betacoronavirus in fruit bats in Sri Lanka and Singapore in recent years. The discovery of lineage D betacoronavirus in guano collected from *Pteropus medius* bats indicates the presence of these viruses in bats, which roost in public spaces like botanic gardens. Thus, it is speculated that Bats belonging to different feeding guilds have an effect on the dynamics and structure of ecosystems, according to the variations in the composition of their excrement. Diversity of fecal Microbial Communities in different *Pteropus* species is given in Table 4.

**Table 4:** Mycobiome, virome, and fecal microbiome across *Pteropus* bat species.

Pteropus species	Bacteria	Fungi	Viruses	References
*P. giganteus*	*Acaligens, Azotobacter, Bacillus, Bartonella, Corynebacterium, Klebsiella, Listeria, Nitrosomonas, Nocardia, Pseudomonas, Salmonella, Streptomycete*	*Alternaria, Aspergillus, Candida, Chrysosporium, Cryptococcus, Exophiala, Fusarium, Histoplasma, Penicillium, Saccharomyces*, *Scopulariopsis*, *Trichophyton*	*Paramyxoviridae*	Juman et al. (2025)
*P. poliocephalus*	*Acinetobacter, Citrobacter, Clostridia, Cornobacter, Escherichia, Klebsiella, Salmonella, Shigella, Streptococcus*	Specific dominant families/genera in feces are not uniformly reported	*Betacoronavirus, Sapovirus, Retrovirus*	Van Brussel et al. (2022)
*Pteropus spp*. (General)	*Proteobacteria, Firmicutes*	Data often limited to opportunistic pathogens isolated from guano	*Coronaviridae, Caliciviridae, Retroviridae, Paramyxoviridae* (e.g., Hendra/Nipah‐mainly urine/saliva but may be present in feces).	F. K. McDougall and Power (2025)

## 6 Connecting Nipah Virus, Bat Microbiota, and AMR Under One Health

### 6.1 Integrated Spillover and Resistome Model

Understanding NiV spillover demands shifting beyond a single‐pathogen viewpoint to a systems view that encompasses bat ecology, the bat‐associated microbiota (bacteriome/resistome), and human‐driven environmental change. Fruit bats of the genus *Pteropus* (particularly *P. medius* in South Asia) are established reservoirs of NiV and display seasonal patterns of infection and shedding that are intimately connected to bat population dynamics, immunological cycles, and environmental variables such as food availability and land‐use change. These ecological drivers that modulate viral dynamics also shape bat microbiomes and the environmental distribution of bacteria and ARGs, creating overlapping pathways by which zoonotic viruses and AMR elements can reach people, domestic animals, and environmental reservoirs (Epstein et al. 2020; McKee et al. 2021). Bats host different bacterial populations across body compartments (gut, oral cavity, skin, urine/urine‐contaminated roost substrates). Recent meta‐reviews and targeted studies show that bat microbiota composition varies with diet, roosting ecology, seasonality, and anthropogenic disturbance; moreover, surveys repeatedly recover bacteria carrying clinically relevant resistance phenotypes and ARGs, indicating bats can carryand in some ecological contexts acquirecomponents of the environmental resistome. These findings do not imply bats are key drivers of the global AMR epidemic, but they do indicate bats contribute to local resistome networks where ecological overlap with people or domestic animals exists (Federici et al. 2022; Devnath et al. 2023). Mechanistically, ARGs move across bacterial communities through horizontal gene transfer (HGT) mediated by plasmids, transposons, and bacteriophages; environmental routes (e.g., contaminated water, soil, food substrates, or fomites contaminated by bat excreta) provide interfaces where HGT can occur between wildlife‐associated bacteria and commensal or pathogenic bacteria of humans and livestock. Untreated wastewater, agricultural antibiotic usage, and livestock manure are examples of anthropogenic inputs that boost environmental selective pressure for resistance and raise the likelihood that bacteria associated with wildlife would share ARGs with bacteria associated with humans (Kim and Cha 2021b; Shang et al. 2025).

Importantly, interactions between viral infection and bacterial ecology in bats may be bidirectional: immunological stressors (nutritional stress, pregnancy, environmental disturbance) that increase viral recrudescence and shedding can also alter host microbiota composition and bacterial shedding patterns, thereby synchronizing windows of increased viral and bacterial/ARG exposure at the human–animal–environment interface. Field research after human NiV cases have discovered viral RNA in roost urine and established temporal clustering of virus detection with specific roost and ambient conditions settings during which interaction with bat excreta by people or domestic animals becomes more likely. The simultaneous or sequential spread of viral and bacterial/ARG risks across interfaces is made possible by this ecological co‐occurrence. (Epstein et al. 2020) A One Health framing therefore, requires integrated surveillance and risk assessment that jointly measures: (i) viral presence and dynamics (NiV RNA/serology), (ii) bat and environmental bacteriomes/resistomes (culture + metagenomics/shotgun sequencing for ARG detection), and (iii) anthropogenic drivers and contact pathways (land use, fruiting/harvest practices, livestock location, sanitation). Systems and network models that incorporate these datasets can identify places where spillover risk and ARG exchange potential coincide, prioritize intervention points (behavioral, ecological, environmental sanitation), and inform cross‐sectoral mitigation measures (Arnold et al. 2024; Aslam et al. 2021).

### 6.2 Converging Risk Hotspots

**Figure 5:**
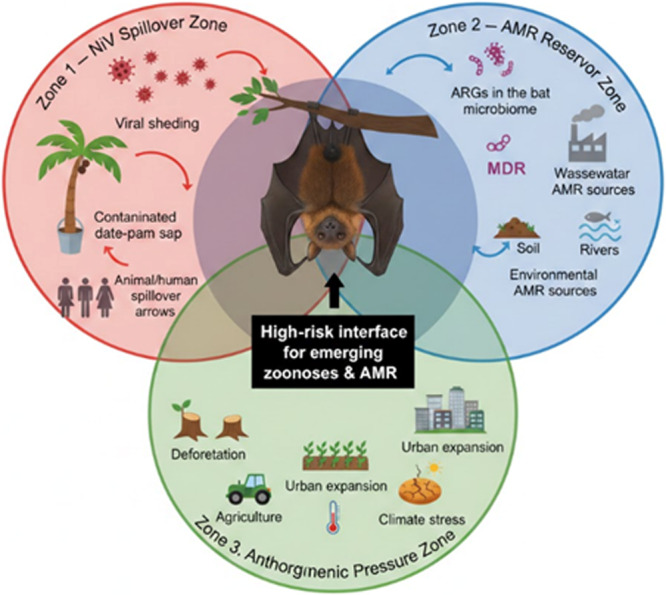
This figure presents a model highlighting the high‐risk interface for the emergence of new zoonotic diseases and Antimicrobial Resistance (AMR), using the flying fox (bat) as the central reservoir. The risk is defined by the critical intersection of three zones: Zone 1 (NiV Spillover Zone), which illustrates the pathway for viruses like Nipah to jump from bats to humans via contaminated resources like date‐palm sap; Zone 2 (AMR Reservoir Zone), which identifies the sources of antibiotic‐resistant genes (ARGs) found in the bat microbiome and environmental pools like wastewater and soil; and Zone 3 (Anthropogenic Pressure Zone), which includes human‐driven activities like deforestation, urban expansion, and climate stress. The simultaneous presence and interaction of these three factors create the optimal conditions for new infectious threats to emerge and for existing antibiotic resistance to spread into human and animal populations.

Certain landscapes and human activities act as ecological “mixing bowls” where NiV exposure risk and AMR transmission potential overlap. In Bangladesh, the winter sap‐harvesting season is repeatedly linked to human NiV outbreaks because fruit bats (*Pteropus* spp.) frequently lick and urinate on the shaved surface of date palms and on collection pots; observational and intervention studies have shown that raw sap consumption is a major risk factor for spillover and that simple physical barriers can reduce bat access to sap. These same collection sites concentrate bat excreta on tree surfaces and nearby ground, creating microenvironments where bat‐associated bacteria and ARGs could be deposited on foodstuffs and fomites that people handle or ingest (Khan et al. 2012; McKee et al. 2021). Bats and humans frequently come into contact with small orchards and backyard fruit trees (sharing feeding platforms, dropped fruit, fruit handled for sale or consumption). Roosts placed within or next to human settlements increase the possibility that bat‐shed viruses and bacteria (including ARG‐bearing taxa) are deposited in sites where humans and domestic animals interact, magnifying potential for cross‐domain microbial contact. Field mapping in Bangladesh reveals that many *P. medius* roosts occur in heavily inhabited regions, underlining this overlap (McKee et al. 2022). Flooding and irrigation that mixes untreated municipal or hospital wastewater with crop fields creates strong selection and dissemination pathways for ARGs. Floodwaters can transport ARGs and resistant bacteria into agroecosystems where bats forage or where fruit and vegetables become contaminated; conversely, contaminated soils and standing water may facilitate contact between environmental bacteria and bacterial strains that colonize bats, domestic animals, or people. Recent metagenomic work on floodwaters in Bangladesh documents ARGs and clinically relevant taxa in post‐flood environments, illustrating how hydrological events create AMR hotspots that intersect with bat foraging ranges (Rahman et al. 2025; Thornber et al. 2022). In peri‐urban and urban situations, *Pteropus* roosts adjacent to marketplaces, food‐handling facilities, or healthcare institutions, where wildlife excreta are placed in proximity to dense human activity and to locations yielding antibiotic residues or resistant bacteria (hospital effluent, wastewater). Such proximity enhances the probability that environmental reservoirs of ARGs would mix with bat‐associated bacterial communities, and that contaminated environmental surfaces enter human exposure pathways (food sold at markets, street vendors, wastewater runoff). Surveillance study has detected NiV RNA in roost urine after spillover episodes, and many studies have shown bats carry antibiotic‐resistant isolates (or ARGs)together these patterns make urban roosts plausible “mixing bowls” (McKee et al. 2022). Taken together, these zones are not isolated hazards: they are coupled by shared factors (land‐use change, poor sanitation, informal food economies, seasonal hydrology) that concurrently increase potential for NiV spillover and for environmental amplification and exchange of ARGs. Operationally, that means surveillance and interventions targeted to these hotspots can provide significant returns for both zoonotic disease prevention and AMR risk reduction. (McKee et al. 2021) The convergence of viral spillover, environmental AMR sources, and escalating anthropogenic pressures creates a critical high‐risk interface for future pandemic threats, as is shown in Figure 5.

### 6.3 Implications for Public Health and Wildlife Conservation

Public‐health and veterinary AMR surveillance platforms should combine animal and environmental sampling at known hotspots (sap sites, orchards, floodplains, urban roosts). The discovery of co‐occurrence patterns required to evaluate joint risk and mechanistic connections is made possible by parallel viral (NiV) and bacterial/ARG testing, which optimally combines targeted PCR, culture + AST, and shotgun metagenomics. Integrated surveillance is suggested by international One‐Health AMR agendas but remains operationally constrained in many LMIC (Low‐ and Middle‐Income Countries) settings; strengthening it in South Asia is both feasible and high‐impact (Delpy et al. 2024). Measures that lessen environmental AMR selection pressure include better sanitation, wastewater treatment, safe disposal of hospital effluent, and stewardship of antimicrobials in livestock) can be combined with interventions that have been shown to reduce NiV spillover (e.g., physical sap barriers, community education to avoid raw sap). Pairing promotes conservation objectives while reducing human exposure and prevents unfavorable outcomes such as habitat destruction that drives bats into new areas controlled by humans. This integrated strategy is supported by data from One‐Health AMR policy assessments and behavioral intervention studies (Khan et al. 2012). Because hotspots are rooted in livelihoods (sap harvest, orchard fruits, smallholder agriculture, market trading), interventions require culturally appropriate engagement that preserves incomes while modifying high‐risk practices. Community‐led adjustments (e.g., sap‐cup covering, fruit cleanliness, scheduling of harvests) are anticipated to lower both virus and bacterial dangers if accompanied by improvements in local sanitation and information about antibiotic use. Field studies from Bangladesh and programmatic One‐Health recommendations underline the relevance of regionally adapted messaging (Yeasmin et al. 2025). While avoiding deadly control methods that endanger bat populations and ecological services (pollination, seed dispersal), roost habitat preservation and reducing anthropogenic pressures that push bats into closer contact with people (loss of foraging habitat, fragmentation) can reduce repeated human‐bat contact.

## 7 Research Gaps and Future Directions

This review highlights key research gaps and future directions regarding the role of *Pteropus medius* in the environmental dissemination of AMR and NiV cross‐species transmission. Despite increasing reports of antimicrobial resistance genes and resistant bacteria in bats, the contribution of *P. medius* in South Asia to environmental AMR circulation remains insufficiently characterized. Current evidence underscores the need for integrated, high‐resolution studies combining microbiome, resistome, and viral surveillance to better understand this interface within a broader One Health and Sustainable Development Goals (SDGs) framework.

A major limitation is the lack of comprehensive metagenomic characterization of the *P. medius* microbiome and resistome. Also, shotgun metagenomics and long‐read sequencing approaches targeting plasmids and other mobile genetic elements remain scarce. Consequently, baseline resistome profiles for this species are not well established, limiting our ability to assess its role in maintaining and disseminating clinically relevant ARGs in the environment (Soto‐López et al. 2025; Devnath et al. 2023). Methodological inconsistencies across studies further restrict comparability, highlighting the need for standardized sampling strategies, laboratory protocols, and bioinformatic workflows aligned with global One Health surveillance priorities.

The absence of longitudinal studies represents a major constraint in the current understanding of bat‐associated AMR, as most available evidence is derived from cross‐sectional snapshots that capture resistome composition at a single point in time. This approach precludes assessment of temporal stability and seasonal variability in ARGs, which are likely influenced by ecological fluctuations such as rainfall patterns, host migration, reproductive cycles, and changes in food availability. This methodological limitation also constrains inference about transmission directionality. Consequently, cross‐sectional sampling cannot determine whether bats acquire resistant bacteria from contaminated environments or whether they contribute to onward dissemination in a sustained ecological cycle. Therefore, conclusions regarding bats as active drivers of AMR spread remain speculative in the absence of longitudinal or experimental studies. In addition, the interpretation of bats as direct sources of antimicrobial resistance requires reconsideration in light of their ecological context. Many bat species included in current studies, particularly those inhabiting urban and peri‐urban areas, are continuously exposed to anthropogenic waste streams, including hospital wastewater, livestock effluents, and agricultural runoff. These sources are well‐established reservoirs of clinically important ARGs. Therefore, bats are more accurately described as environmental sentinels or secondary hosts that acquire, transiently harbor, and potentially redistribute resistant bacteria rather than primary evolutionary sources of resistance determinants.

Evidence suggests that ecological factors such as breeding cycles, migration, nutritional stress, and habitat disturbance may influence both microbiome composition and NiV shedding dynamics. However, longitudinal studies remain lacking. Repeated sampling across seasons and ecological contexts is therefore essential to determine whether shifts in microbial communities are associated with changes in viral shedding intensity or ARG abundance. Importantly, such ecological dynamics are increasingly shaped by land‐use change, deforestation, and biodiversity loss. Integrated analyses combining metagenomics with viral detection (PCR and serology) and host immune profiling would further clarify whether specific microbiome configurations are associated with altered viral shedding or increased environmental release of ARGs (Devnath et al. 2023; Thornber et al. 2022; F. McDougall et al. 2019).

A further critical gap concerns the lack of mechanistic evidence for ARG transmission from bats to bacteria infecting humans or livestock. Although environmental detection of ARGs has been widely reported, the pathways underlying their dissemination remain poorly defined. Mechanistic studies investigating horizontal gene transfer, including plasmid‐mediated conjugation, bacteriophage transduction, and integron‐associated gene capture, are urgently needed. These should be supported by culture‐based bacterial isolation, whole‐genome sequencing, and comparative genomic analyses to trace ARG movement across environmental and host compartments. Strengthening such a mechanistic understanding is essential for evidence‐based policymaking through interdisciplinary collaboration. Besides, the detection of both Nipah virus and antimicrobial‐resistant bacteria within the same ecological settings does not provide evidence of direct interaction, shared transmission pathways, or mechanistic association between these entities. Instead, their co‐presence likely reflects overlapping exposure to common environmental drivers, particularly anthropogenically impacted habitats influenced by human activity, livestock production, and wastewater contamination. At present, there is limited empirical evidence demonstrating direct transfer of antimicrobial resistance determinants from bats to human pathogens or coordinated transmission between viral and bacterial agents within bat‐associated systems. Therefore, interpretations suggesting linked dissemination of Nipah virus and antimicrobial resistance should be treated with caution, and future studies should employ integrated virological, microbiological, and genomic approaches to disentangle true transmission pathways from ecological co‐occurrence.

Finally, operational One Health surveillance systems integrating NiV monitoring, environmental resistome profiling, and exposure assessment in humans and livestock remain limited in South Asia. Establishing sentinel surveillance sites at high‐risk interfaces such as date palm sap collection areas, peri‐domestic orchards, urban bat roosts, and flood‐prone agricultural regions is crucial. In parallel, environmental studies focusing on hydrological and ecological dissemination pathways, particularly during flooding events, will be essential to understand large‐scale ARG spread. Coordinated regional frameworks, standardized methodologies, and integrated ecological modeling are needed to translate surveillance data into effective public health interventions (Delpy et al. 2024; Directorate General of Health Services DGHS 2020).

From a broader systems perspective, embedding these strategies within the 2030 Agenda for Sustainable Development is essential. Environmental degradation, including deforestation, habitat fragmentation, and illegal wildlife trade, increases biodiversity loss and promotes closer human–wildlife contact, thereby elevating zoonotic spillover risks. These drivers are tightly linked to the emergence of both AMR and NiV and underscore the need for intersectoral collaboration across environmental, veterinary, and human health domains. Strengthening governance, raising awareness, and implementing evidence‐based management practices remain critical for controlling AMR dissemination and reducing zoonotic disease emergence (Kaddeche et al. 2026).

Effective disease surveillance and control programs are essential for early detection of emerging pathogens and prevention of spillover events. In many low‐resource settings, habitat disruption has already been shown to increase human contact with wildlife, contributing to rising zoonotic disease incidence. Therefore, proactive, upstream surveillance systems—integrating ecological monitoring, pathogen detection, and community engagement are vital to identify risks before they escalate. Such integrated approaches not only reduce the likelihood of disease emergence but also support sustainable health outcomes consistent with SDG 3, SDG 15, and broader One Health principles.

## 8 Conclusion

At the nexus of humans, wildlife, and shared ecosystems, *Pteropus medius* serve as an important ecological connector linking Nipah virus spillover dynamics with environmental antimicrobial resistance (AMR) pathways. The evidence compiled in this study shows that viral spillover is the outcome of interrelated ecological, behavioral, and anthropogenic factors that influence the timing and location of human exposure rather than a distinct virological event. Simultaneously, the *P. medius* fecal microbiota, thought to be peripheral to spillover dynamics, emerges as a complex reservoir with a variety of bacterial communities capable of harboring and possibly spreading antimicrobial resistance genes (ARGs). In this context, bat roosting and foraging sites may function as ecological convergence zones where viral, bacterial, and environmental transmission pathways intersect, reinforcing the relevance of an integrated One Health surveillance approach. An effective method to simultaneously monitor Nipah virus circulation and environmental resistomes is targeted surveillance at converging hotspots, such as date‐palm sap collection locations, peri‐domestic orchards, flood‐affected agricultural regions, and urban roosts. To ascertain whether and by what processes ARGs migrate between animal, environmental, and human bacterial communities, it will be crucial to integrate viral diagnostics with shotgun metagenomics, plasmid/resistome mapping, and culture‐based antimicrobial susceptibility testing. concurrently lower spillover risk and AMR selection pressure without jeopardizing bat conservation or local livelihoods.

Overall, the One Health framework highlights the need for coordinated, multidisciplinary collaboration across the medical, veterinary, environmental, and wildlife health sectors to understand better and mitigate these interconnected risks. In the future, culturally relevant behavioral and environmental interventions, along with genomics‐enabled monitoring, can improve early detection, clarify transmission dynamics, and inform sustainable disease prevention strategies while preserving ecological balance and supporting wildlife conservation.

## Author Contributions

Punam Chowdhury (First Author) conceptualized the manuscript, prepared and edited the initial draft. Shah Md. Tanvir Khan and Sajal Roy (Co‐author) prepared the initial draft and edited the manuscript. Md. Shohel Al Faruk (Corresponding Author) provided overall supervision and guidance. All authors contributed to the discussion, reviewed all figures and tables, and approved the final version of the manuscript.

## Funding

The authors have nothing to report.

## Ethics Statement

The authors have nothing to report.

## Consent

All authors read and approved to publish this manuscript.

## Conflicts of Interest

The authors declare no conflicts of interest.

## Generative AI and AI‐Assisted Technologies in the Writing Process

During the preparation of this manuscript, tools such as Quill Bot, Grammarly, ChatGPT, and Gemini AI were used to polish the language, correct grammar, and generate Figure 5. After using these tools, the authors carefully reviewed and edited the content as necessary, and they take full responsibility for the final content of the publication.

## Data Availability

No new data were generated or analyzed in this study. All data discussed in this review are derived from previously published studies, which are appropriately cited within the article.
